# Neurophysiological Correlates of Featural and Spacing Processing for Face and Non-face Stimuli

**DOI:** 10.3389/fpsyg.2017.00333

**Published:** 2017-03-13

**Authors:** Marcello Negrini, Diandra Brkić, Sara Pizzamiglio, Isabella Premoli, Davide Rivolta

**Affiliations:** ^1^School of Psychology, University of East LondonLondon, UK; ^2^Department of Economics (AE1), School of Business and Economics, Maastricht UniversityMaastricht, Netherlands; ^3^Aston Brain Centre, School of Life and Health Sciences, Aston UniversityBirmingham, UK; ^4^School of Architecture, Computing and Engineering, University of East LondonLondon, UK; ^5^Institute of Psychiatry, Psychology and Neuroscience, King’s College LondonLondon, UK

**Keywords:** face perception, object perception, holistic processing, configural processing, EEG, P100, N170

## Abstract

The peculiar ability of humans to recognize hundreds of faces at a glance has been attributed to face-specific perceptual mechanisms known as holistic processing. Holistic processing includes the ability to discriminate individual facial features (i.e., featural processing) and their spatial relationships (i.e., spacing processing). Here, we aimed to characterize the spatio-temporal dynamics of featural- and spacing-processing of faces and objects. Nineteen healthy volunteers completed a newly created perceptual discrimination task for faces and objects (i.e., the “University of East London Face Task”) while their brain activity was recorded with a high-density (128 electrodes) electroencephalogram. Our results showed that early event related potentials at around 100 ms post-stimulus onset (i.e., P100) are sensitive to both facial features and spacing between the features. Spacing and features discriminability for objects occurred at circa 200 ms post-stimulus onset (P200). These findings indicate the existence of neurophysiological correlates of spacing vs. features processing in both face and objects, and demonstrate faster brain processing for faces.

## Introduction

Humans can typically recognize hundreds of faces with ease. It has been suggested that this extraordinary ability relies on face-specific perceptual processing that allows the recognition of (upright) faces as a gestalt or a global representation (Rossion, 2008). This perceptual processing has been referred to as “holistic” (Tanaka and Farah, 1993), “configural” (Maurer et al., 2002), or “second-order relational” (Diamond and Carey, 1986). Despite the different terminology adopted, *holistic* processing (which is the term we adopt here) refers to the simultaneous (i.e., parallel) processing of multiple facial features (e.g., eyes, mouth, and nose – *featural processing*), and their metric distance (e.g., inter-ocular distance or nose-mouth distance – *spacing processing*) (see McKone and Yovel, 2009; Piepers and Robbins, 2012 for reviews on the subject). Object perception (even objects of expertise; Robbins and McKone, 2007), on the other side, specifically relies on featural processing only (Biederman, 1987; Tanaka and Simonyi, 2016).

Holistic face processing has been assessed using different behavioral paradigms. For instance, face perception is negatively affected by stimulus inversion (i.e., *face-inversion effect*), a manipulation believed to disrupt holistic processing (Yin, 1969). In the composite-face task (Young et al., 1987), identifying the top-halves of faces is harder when aligned with competing-identity bottom halves (forming the illusion of a new face) compared to when the halves are misaligned (i.e., *composite-face effect*). Despite widespread use of the “face-inversion” and the “composite-faces” to probe holistic face processing in typical and atypical populations (Palermo et al., 2011; Rossion et al., 2011; Rivolta et al., 2012b), these tasks do not directly manipulate facial features and their spacing relationship. Experimental paradigms that allow to manipulate facial features and their spacing relationship include, but are not limited to, the Jane Task (Mondloch et al., 2002) and the Albert Task (Yovel and Kanwisher, 2004). In these identity-matching tasks, participants are asked to decide whether two sequentially presented faces are the same or different, when the facial features (engaging *featural processing*) or the spacing between them (engaging *spacing processing*) differ. Previous results showed that performance in these identity-matching tasks is impaired after stimulus inversion, suggesting that holistic face processing integrates both featural shapes and their spacing (McKone and Yovel, 2009).

Neuroimaging studies have shown that separate and dissociable brain regions mediate spacing and featural processing. For instance, Transcranial Magnetic Stimulation (TMS) studies have demonstrated the involvement of the right lateral occipital lobe (right occipital face area, OFA; Pitcher et al., 2007) and of the left middle frontal gyrus (MFG; Renzi et al., 2013) in featural-face processing. Spacing processing, on the other side, has been related to the activity of the right inferior frontal gyrus (IFG; Renzi et al., 2013). Furthermore, functional magnetic resonance imaging (fMRI) studies have provided evidence of a correlation between the activity in the fusiform gyrus and spacing processing (Maurer et al., 2007)^1^. In summary, causal and correlational evidence from neuroimaging suggests that different face-sensitive regions in the occipital, temporal, and frontal lobe are involved in different aspects of face perception (i.e., featural vs. spacing).

Although, TMS and fMRI provide important evidence about the temporal and spatial features of face perception, they both have some limitations: TMS has restrictions in targeting regions that lie in the ventral surface of the temporal lobe, whereas fMRI has poor temporal resolution (Amaro and Barker, 2006). In contrast to these methods, Event-Related Potentials (ERPs) as measured with the electroencephalogram (EEG) reveal the timing of neuronal events underlying sensory and cognitive processes with millisecond precision across the whole scalp. EEG (along with Magnetoencephalography – MEG) studies suggest that the perception of visual stimuli (e.g., faces and objects) induces a sequence of evoked components within the first 200 ms after stimulus presentation (see Rossion, 2014 for a review). The most investigated component, N170 (M170 when tested with MEG), peaks at around 170 ms post-stimulus onset (Bentin et al., 1996; Liu et al., 2002). The N170 for faces is stronger than for any other visual category tested so far, and appears to be generated by activities of the occipital cortex and the fusiform gyrus (Itier et al., 2007; Rivolta et al., 2012b; Rossion, 2014). An earlier ERPs component, P100, peaking at around 100 ms post-stimulus onset (P100 is a positive component, also known as M100 when recorded with MEG) (Linkenkaer-Hansen et al., 1998; Rivolta et al., 2012a), is believed to reflect low-level features of visual stimuli, such as size and luminosity. Evidence for the face-sensitivity of P/M100 is mixed, with some studies finding face-sensitivity (Rivolta et al., 2012a) and others not (Boutsen et al., 2006). Another positive component, P200, which peaks at around 200 ms post-stimulus onset with a topography similar to P100 (Mercure et al., 2008) has been suggested to reflect cortical visual feedback from high- to low- level visual areas (Kotsoni et al., 2006) and to be involved in emotion face perception (Dennis and Chen, 2007).

EEG/MEG research on the role of early-evoked (100–200 ms) potentials in different aspects of face processing is surprisingly limited. There is indication suggesting early P100 sensitivity for spacing processing (Halit et al., 2000). For example, Wang et al. (2015) showed that, under certain attentional conditions, P100 is larger for spacing- as compared to featural-face processing. These neurophysiological studies, along with behavioral evidence (Zinchenko et al., 2015), suggest that the human visual system can rapidly (∼100 ms) discriminate between featural and spacing facial manipulations. The involvement of N/M170 in holistic face processing has been shown with the face-inversion effect (i.e., N170 is larger and delayed for inverted faces, see Rossion et al., 2000), the composite-face effect (i.e., N170 is larger for aligned than misaligned faces) (Letourneau and Mitchell, 2008) and Mooney faces (Rivolta et al., 2014a). The N/M170, however, is not sensitive to featural vs. spacing modulations of face stimuli (Halit et al., 2000; Scott and Nelson, 2006; Wang et al., 2015). This suggests that holistic processing investigated by tasks tapping into spacing vs. featural differences and holistic processing, as assessed by face inversion and composite face, may occur at different time-scales. Moreover, using featural and spatial modified face stimuli, Mercure et al. (2008) showed a significant effect on the P200 amplitude for faces with a spatial/configural modification, where the amplitude of P200 was reduced by the “feature manipulation” compared to the “spacing manipulation” (Mercure et al., 2008). Wang et al. (2015), however, reported a larger P200 for the featural-face processing using the steady-state visual evoked potentials (SSVEP) to differentiate spacing-vs. featural-face processing.

Overall, the current literature suggests that face-sensitive electrophysiological components may mediate spacing and featural mechanisms. However, it is still unclear whether these effects are face-specific or whether they also characterize the perception of non-face stimuli. In the current study, we investigated the spatio-temporal dynamics of spacing and featural detection in facial and non-facial stimuli with high-density EEG. In the experiment, we implemented a newly created identity-matching task called the “University of East London Face Task” (UEL-FT). This task tests feature and spacing perception for face and non-face stimuli. Based on previous evidence, we predicted to find three early face-sensitive components: P100, N170, and P200. Furthermore, we expected differences between featural and spacing effects for faces in early (i.e., P100 and N170) ERP components, especially in posterior electrodes. Since no previous EEG study specifically targeted featural and spacing processing in non-face stimuli, we did not advance a specific prediction on the spatio-temporal dynamics of house processing.

## Materials and Methods

### Participants

Nineteen participants (12 females) without any recorded history of psychiatric or neurological disorder and with a mean age of 28 years (range 21–41) participated in the experiment. All participants had normal or correct-to-normal vision and did not report everyday life problems in face recognition. The study was performed according to the Declaration of Helsinki and approved by the ethical committees of University of East London (UEL). After complete description of the study to the participants, written informed consent was obtained.

### Stimuli

**FIGURE 1:**
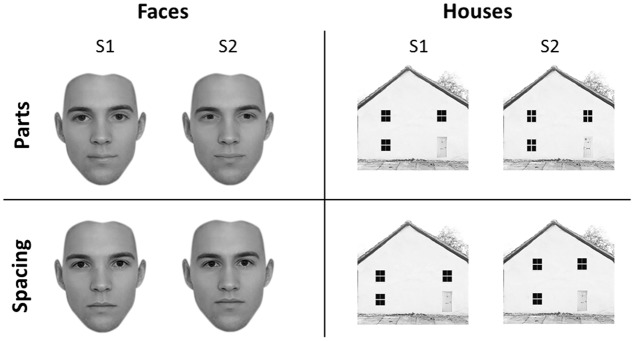
**Experimental stimuli.** Face (left) and house (right) stimuli adopted in the University of East London (UEL) face task. Parts (top) and spacing (bottom) manipulations are shown for both categories. Some of the face pictures have been obtained from www.beautycheck.de.

Forty-five faces and forty-five houses were created using five “original” faces and five “original” houses with a resolution of 300 × 300 pixels, in line with previous studies (Yovel and Kanwisher, 2004). Adobe Photoshop software (Adobe Systems, Inc., San Jose, CA, USA) was used to create the feature and spacing sets for the face and house stimuli. Starting from the original stimuli, which were downloaded from the internet, for both categories we created a *feature set* and a *spacing set*. Each set was made of four variations (**Figure 1**).

#### Face Stimuli

For the feature set, the two eyes and the mouth were replaced with eyes and mouth of similar shape taken from other stimuli (not belonging to the original set) to produce four variations of each of the five “original” faces. For the spacing set, the eyes were shifted inward or outward by 4–5 pixels and the mouth was shifted downward or upward by 4–5 pixels. All faces were cropped to exclude the hair.

#### House Stimuli

For the feature set, four variants of each of the five “original” houses were constructed by replacing windows and doors with windows and doors of similar shape but of different texture. For the spacing set, the location of the windows and doors was shifted so that they were closer together or farther apart and the two top windows were closer to or farther from the roof, on average by 15 pixels.

We did not adopt the original “Albert Task,” or “Jane Task” because they are characterized by fewer stimuli, which are repeated many times during the task. Previous studies suggest that stronger holistic processing is engaged with tasks that adopt many different stimuli (repeated few times), as compared to tasks adopting few stimuli (repeated many times) (McKone and Yovel, 2009).

#### Experimental Design

**FIGURE 2:**
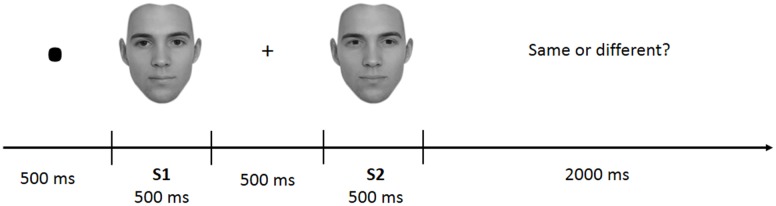
**Example of trial structure**.

The task was divided into four blocks of 100 trials each. Each block included face-parts (FP), face-spacing (FS), house-parts (HP), or house spacing (HS) stimuli. Block presentation was randomized with the constraint that the two face and the two house blocks were presented in sequence (and never alternated). Participants received instructions at the beginning of each block. In each trial a pair of stimuli belonging to the same category (face or house) and condition (feature or spacing) was presented. Each trial started with a fixation mark (500 ms), followed by the first stimulus (S1–500 ms), followed by a fixation cross (500 ms) and the second stimulus (S2–500 ms) (see **Figure 2**). Participants had to judge whether S1 and S2 were identical (i.e., “same” response) or different (i.e., “different” response) by pressing one of two different keys (i.e., left arrow for “same” and right arrow for “different”) on a computer keyboard. In both the spacing and feature conditions half of the trials were “identical” (i.e., S1 was equal to S2) and half were “different” (i.e., variations in features or spacing from S1 to S2). Participants were given 2000 ms time to make a decision; after this time the response was considered as incorrect. They were also instructed to minimize big movements of the head and shoulders, avoid contraction of face muscles and try to blink and swallow in the period between trials.

All stimuli were shown in the center of a CRT monitor (30 cm diameter, 60 Hz refresh rate) installed inside an electrically shielded room, and placed at a distance of ∼100 cm from the participant’s head. Face and house stimuli were presented within a frame that covered a visual angle of 4.8° × 4.8°. The EEG experiment was programmed and delivered with Psych Toolbox (Matlab, MathWorks^®^). Stimuli did not differ in luminance [FP: *M* = 190 cd/cm^2^, SEM = 1.53; FS: *M* = 191 cd/cm^2^, SEM = 1.48; HP: *M* = 187 cd/cm^2^, SEM = 5.7; HS: *M* = 186 cd/cm^2^, SEM = 5.66; *F*(3,79) = 0.31, *p* = 0.82] and are reported in Supplementary Figure 1.

### Behavioral Analysis

Behavioral analysis, accuracy, and reaction times (RTs), was performed in SPSS by means of a repeated measures ANOVA with factors category (face vs. house) and condition (parts vs. spacing). “Identical” (i.e., S1 = S2) and “different” (i.e., S1 ≠ S2) trials have been collapsed and only correct trials have been considered for statistical analysis.

### EEG Data Processing and Statistical Analysis

EEG data were recorded with a high-density 128-channel Hydrocel Geodesic Sensor Net (Electrical Geodesic Inc., EGI, Eugene, OR, USA) referenced to the vertex (Tucker, 1993). The EEG signal was amplified with EGI NetAmps 200, digitized at 500 Hz, band-pass filtered from 0.1 to 200 Hz and stored for offline analysis. Impedance was kept below 50 kΩ. EEG data processing was performed using the open source Matlab toolbox “FieldTrip^2^” (Oostenveld et al., 2011). A band-pass filter (1–60 Hz) and a notch filter (50 Hz) were first applied to, respectively, limit the signal of interest and remove the power line noise. Data were subsequently segmented into epochs (i.e., trials) of 2500 ms length, starting 500 ms before S1 and ending 500 ms after S2. Each trial was baseline-corrected by removing a period of 400 ms (from 500 to 100 ms) before S1, during which subjects were at rest between trials. Therefore, both S1 and S2 were referred to the same baseline. Eye-blinks and muscle artifacts were detected using the automatized FieldTrip routine. Noisy electrodes were excluded and their signal substituted by an interpolation of the activity of neighboring electrodes (thus, a total of 128 electrodes per participant were considered in the analyses). After linear interpolation, the EEG signal was re-referenced according to the average activity of the 128 electrodes (Dien, 1998). Correct trials only were considered for all the EEG analysis. The correct average and artifact-free trials for each condition were: FP = 70 (*SD* = 12); FS = 60 (*SD* = 8); HP = 68 (*SD* = 10); HS = 76 (*SD* = 9).

**FIGURE 3:**
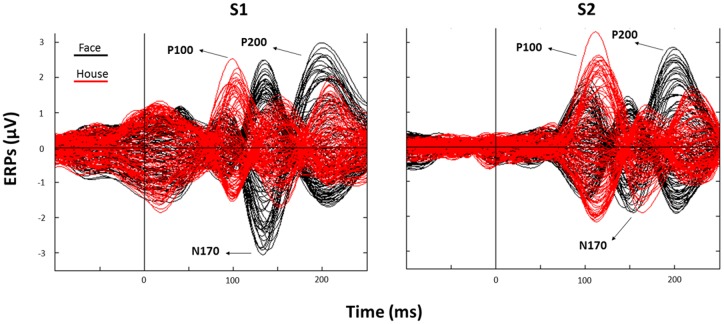
**Corresponding event related potentials (ERPs) traces (butterfly plots) for all electrodes as averaged across all trials (black: Face; red: House) after S1 (left panel) and S2 (right panel) presentation (“0” indicates stimulus onset)**.

**FIGURE 4:**
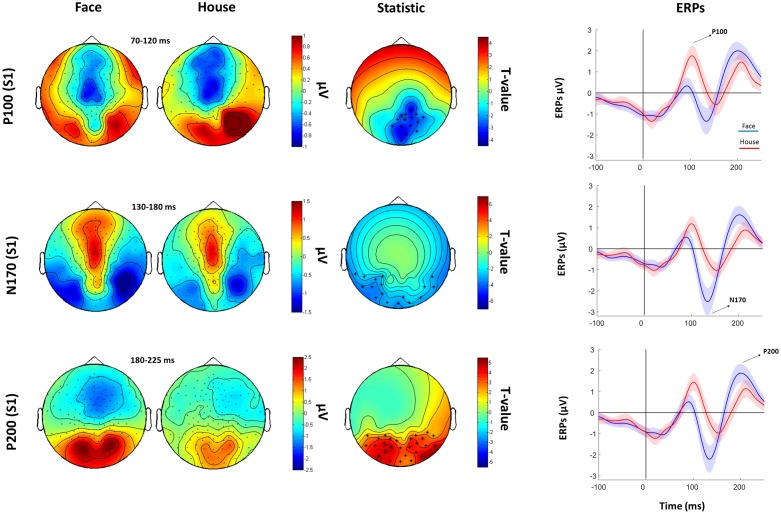
**Faces Vs. House comparison.** Left: Topographical plots for S1-evoked ERP components (P100, N170, and P200) for Face and House conditions. Middle: *t*-statistic maps of the ERP amplitude Face vs. House differences. Crosses indicate significant channels (^∗^: *p* < 0.01). Right grand-averaged ERPs traces for face and houses averaged across statistically significant electrodes (shades represent the SEM).

The subsequent analysis was divided in two parts. First, we aimed to verify the presence, in our data, of traditionally recorded early “face-sensitive” components, such as the P100, N170, and P200. To avoid potential adaptation effects (i.e., S2 amplitude reduction due to S1 perception), this was achieved by means of face-house contrasts on S1. After visual inspection of the grand-average ERPs data (**Figure 3**) and looking at individual peaks, we defined S1 time-windows of interest as follows: P100 (70–120 ms), N170 (130–180 ms), and P200 (180–250 ms) (see **Figure 4** for the topography of the three components). Second, to ascertain whether within-class part-based vs. spacing-based perceptual mechanisms were characterized by different ERPs features, we compared features vs. spacing conditions separately for face and house stimuli (i.e., FP vs. FS and HP vs. HS). This analysis, in line with previous “match-to-sample” EEG studies (Mercure et al., 2008; Eimer et al., 2011), was carried out on S2. Time-windows of interest were determined by visual inspection (**Figure 3**) of the data as follows: P100 (Face: 60–125 ms; House: 65–140 ms), N170 (Face: 125–175 ms; House: 140–190 ms), P200 (Face: 175–250 ms; House: 190–250 ms). In both spacing and feature conditions half of the trials were “identical” (i.e., the stimulus was repeated) and half were “different” (i.e., variations in features or spacing). The EEG analysis, as in previous studies that adopted a similar task (Yovel and Kanwisher, 2004; Pitcher et al., 2007), has been conducted collapsing same and different trials together and considering only correct trials.

In order to analyze sensor-level EEG data we adopted the approach from our previous M/EEG studies (Premoli et al., 2014; Rivolta et al., 2015), by using a non-parametric cluster-based permutation analysis (Maris and Oostenveld, 2007) on each electrode separately for the P100, N170, and P200 components. Specifically, a paired *t*-test was conducted for each electrode at each time bin within the P100, N170, and P200 time-windows. T-values exceeding an *a priori* threshold of *p* < 0.01 were clustered based on adjacent time bins and neighboring electrodes. Cluster-level statistics were calculated by taking the sum of the *t*-values within every cluster. The comparisons were done with respect to the maximum values of summed *t*-values. By means of a permutation test (i.e., randomizing data across conditions and rerunning the statistical test 1500 times), we obtained a reference distribution of the maximum of summed cluster *t*-values to evaluate the statistic of the actual data. Clusters in the original dataset were considered to be significant at an alpha level of 5% if <5% of the permutations (*N* = 1500) used to construct the reference distribution yielded a maximum cluster-level statistic larger than the cluster-level value observed in the original data.

Since previous studies showed a prominent role of posterior (i.e., occipito-temporal) electrodes in detecting face-sensitive activity (Rossion, 2014), we ran the first analysis (S1) on posterior sensors only (*N* = 41) in order to increase the sensitivity of the statistics. However, due to the lack of any *a priori* predictions about the location from where potential conditions effect could arise, and since previous fMRI and TMS research highlighted spacing vs. feature activity even in the frontal cortex (Maurer et al., 2007; Renzi et al., 2013), within-category features vs. spacing contrasts were performed on all the 128 electrodes.

## Results

### Behavioral Results

Analysis of accuracy revealed a main effect of category [*F*(1,18) = 33.80, *p* < 0.001], with faces (*M* = 77.6%; SEM = 1.9) showing worse accuracy than houses (*M* = 86.2%; SEM = 1.3). There was no main effect of condition [*F*(1,18) = 2.3, *p* = 0.12], albeit there was a statistically significant category × condition interaction [*F*(1,18) = 65.25, *p* < 0.001]. Pairwise comparison (Bonferroni corrected) showed that our participants were more accurate in the FP (*M* = 84.0%; SEM = 2.2) condition than in FS (*M* = 71.2%; SEM = 2.0) (*p* < 0.001), whereas were less accurate in the HP (*M* = 82.0%; SEM = 1.7) than HS (*M* = 90%; SEM = 1.6) (*p* < 0.001).

Analysis of RTs (correct trials only) showed no main effect of condition [*F*(1,18) = 1.94, *p* = 0.18] and no main effect of category [*F*(1,18) = 1.17, *p* = 0.30]. There was, however, a category × condition interaction [*F*(1,18) = 5.54, *p* = 0.03]. Follow-up comparisons (Bonferroni corrected) showed that FS (*M* = 1124 ms, SEM = 107) was characterized by longer RTs than FP (*M* = 910 ms, SEM = 56) (*p* = 0.047).

### ERPs Results

#### Face vs. House Contrasts

Cluster-based permutation analysis of the P100 showed that faces had reduced amplitude than houses in a cluster of 15 electrodes. The N170 was more negative for faces than for houses in a cluster of 13 electrodes. The P200 for faces was stronger than for houses in a cluster of 14 electrodes (**Figure 4**).

#### Within-Class Features vs. Spacing Contrasts

**FIGURE 5:**
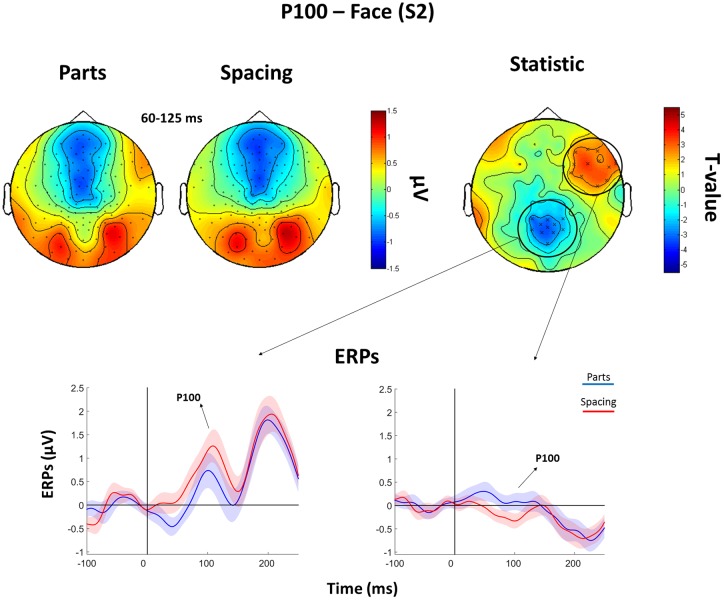
**Features (FP) vs. spacing (FS) contrasts for the P100 as elicited by S2 faces.** Top: Topographical plots of grand-averaged ERPs for the two Face conditions (Parts and Spacing), *t*-statistic map distribution (X: *p* < 0.05). Bottom: Grand-averaged ERPs traces as averaged across statistically significant electrodes (shades represent the SEM).

**FIGURE 6:**
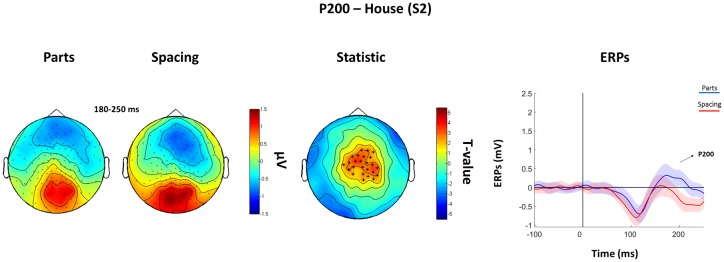
**Features (HP) vs. spacing (HS) contrasts for the P200 as elicited by S2 houses.** Topographical plots of grand-averaged ERPs for the two House conditions (Parts and Spacing) (left), *t*-statistic map distribution (^∗^: *p* < 0.01) (middle), and grand-averaged ERPs traces as averaged across statistically significant electrodes (right) (shades represent the SEM).

The analysis of the P100 for *faces* showed that FS led to higher P100 amplitude in a parietal-occipital cluster of 11 electrodes. Contrary, FP was characterized by higher P100 amplitude over a cluster of 12 right fronto-temporal electrodes (**Figure 5**). Given the dipole shape and location and taking into account the relatively poor spatial EEG resolution, it is likely that this spatial dissociation is due to the same (occipital) dipole. No FP vs. FS differences were found in the N170 and P200 (all *P*s > 0.05). The analysis of the P200 for *houses* showed higher amplitude for HP than HS over a fronto-parietal (i.e., central) cluster including 24 electrodes (**Figure 6**). No HP vs. HS differences were found for the P100 and N170 (all *P*s > 0.05).

## Discussion

The current study investigated the ERPs markers (P100, N170, and P200) of featural and spatial processing in face and non-face visual stimuli. We implemented a newly developed perceptual discrimination task (i.e., UEL-FT) to demonstrate that facial and non-facial featural vs. spacing processing displays different spatio-temporal dynamics. Results, in line with our hypotheses, demonstrate that the human visual system can discriminate spacing vs. featural manipulations as early as after 100 ms post-stimulus onset (P100) for faces; whereas it requires circa 200 ms (P200) to discriminate spacing vs. featural manipulations for house stimuli.

At the behavioral level, participants were faster and more accurate at recognizing featural manipulations. This is in line with previous studies (Le Grand et al., 2006; Rivolta et al., 2012b; Tanaka et al., 2014; Wang et al., 2015) demonstrating how facial features are easier to process than manipulations of the distance between them. In the ERPs analysis, we showed that the early component P100 is sensitive to manipulations of faces in the UEL-FT task, which is in line with the literature (Mercure et al., 2008). Furthermore, our findings point out differences in the P100 amplitude distribution on the scalp, with parietal-occipital electrodes showing prominent spacing activity for faces, and with fronto-temporal electrodes showing prominent featural activity for faces.

Face perception is mediated by a network of cortical and subcortical brain regions (Haxby et al., 2000; Rivolta et al., 2014b). It has been demonstrated that some face areas are mainly involved in specific aspects of face processing. For instance, TMS delivered at circa 100 ms post-stimulus onset showed that right-OFA and left-MFG are implicated in featural processing, whereas the right IFG is involved in spacing processing (Pitcher et al., 2007; Renzi et al., 2013). Previous EEG and MEG studies showed occipital and frontal face-sensitive activity at the same latency (P/M100) (Linkenkaer-Hansen et al., 1998; Halgren et al., 2000; Rivolta et al., 2012a, 2014a). These findings need to be taken into careful consideration since the spatial (occipital vs. frontal) EEG difference may have its origin in the same (likely occipital) dipole. Mainly, our findings of P100 discriminability between spacing and feature face manipulations confirms previous evidence of early face-sensitive processing (Halit et al., 2000; Wang et al., 2015), suggesting that the visual system is sensitive to featural and spacing manipulations as early as 100 ms post-stimulus onset.

Critically, house stimuli that underwent the same manipulations of face stimuli did not show a P100 effect. This aligns with previous literature demonstrating that TMS over the OFA at ∼100 ms only affected spacing processing for faces, but not for houses (Pitcher et al., 2007), thus pointing toward face-specific perceptual mechanisms at 100 ms post-stimulus onset. Neurophysiological activity that discriminated between features and spacing processing for house stimuli was evident at the P200 level, suggesting that face processing occurs earlier that object processing (Farah et al., 1998; Crouzet et al., 2010).

In line with previous studies (Halit et al., 2000; Mercure et al., 2008; Wang et al., 2015), we did not find significant condition effects for faces at the level of the N170. Previous evidence suggests that the composite-face effect (Letourneau and Mitchell, 2008) and the inversion effect (Rossion et al., 2000) affect the N170 amplitude (but not, or to a lesser extent, the P100), indicating a critical involvement of this ERP component in holistic face processing (see Yovel, 2015 for a recent review). These differences between P100 and N170 indicate that holistic processing investigated by spacing vs. featural differences (McKone and Yovel, 2009), and holistic processing assessed by other types of tasks (i.e., face inversion or composite face) may occur at different time-scales. Our results, along with previous neuroimaging (fMRI, TMS) findings (Mercure et al., 2008; Wang et al., 2015; Yovel, 2015; Zachariou et al., 2016), contribute to define the EEG temporal dimension of early face processing. The visual system is able to discriminate the facial features and their distance or “spacing distribution,” as early as the P100 occurs and that might be later (N170) integrated into forming an holistic representation. Finally, we did not report features vs. spacing face differences in the P200, which supports the idea that this component might reflect emotional salience processing (i.e., P200 is greater for negative emotional faces and pictures) (Cuthbert et al., 2000; Dien et al., 2004). Furthermore, confirming previous findings, we detected face-sensitive N170 (Bentin et al., 1996) and P200 (Boutsen et al., 2006) activity. The P100 was more positive for houses than faces, potentially indicating that low-level features may differentiate face and house stimuli of our experiment (albeit luminance and size were similar between categories). Notwithstanding, we believe that this effect should not undermine the validity of our main findings, which are within-category.

## Conclusion

Our EEG and behavioral findings suggest that featural vs. spacing processing for faces occurs at ∼100 ms (P100), whereas it occurs at ∼200 ms (P200) post-stimulus onset for houses. These results have important implications for theories of holistic face processing and their neurophysiological correlates. Future studies should try to implement EEG source connectivity approaches to further describe the spatial-dynamics of spacing and featural neural processing and characterize the topology of the evoked activity.

## Author Contributions

MN, DB, and DR contributed to the design, recordings, analyses, and write-up. IP and SP contributed to analyses and write-up of the study.

## Conflict of Interest Statement

The authors declare that the research was conducted in the absence of any commercial or financial relationships that could be construed as a potential conflict of interest.

## Footnotes

1 Note that Maurer et al. (2007) report fMRI activity in the fusiform gyrus, but outside the functionally localized FFA.

2 http://fieldtrip.fcdonders.nl/
