# Supplementary figures and images for: Neurophysiological Correlates of Featural and Spacing Processing for Face and Non-face Stimuli
Source: Front Psychol. 2017 Mar 13;8:333. doi: 10.3389/fpsyg.2017.00333 (PMC5346548; doi:10.3389/fpsyg.2017.00333)

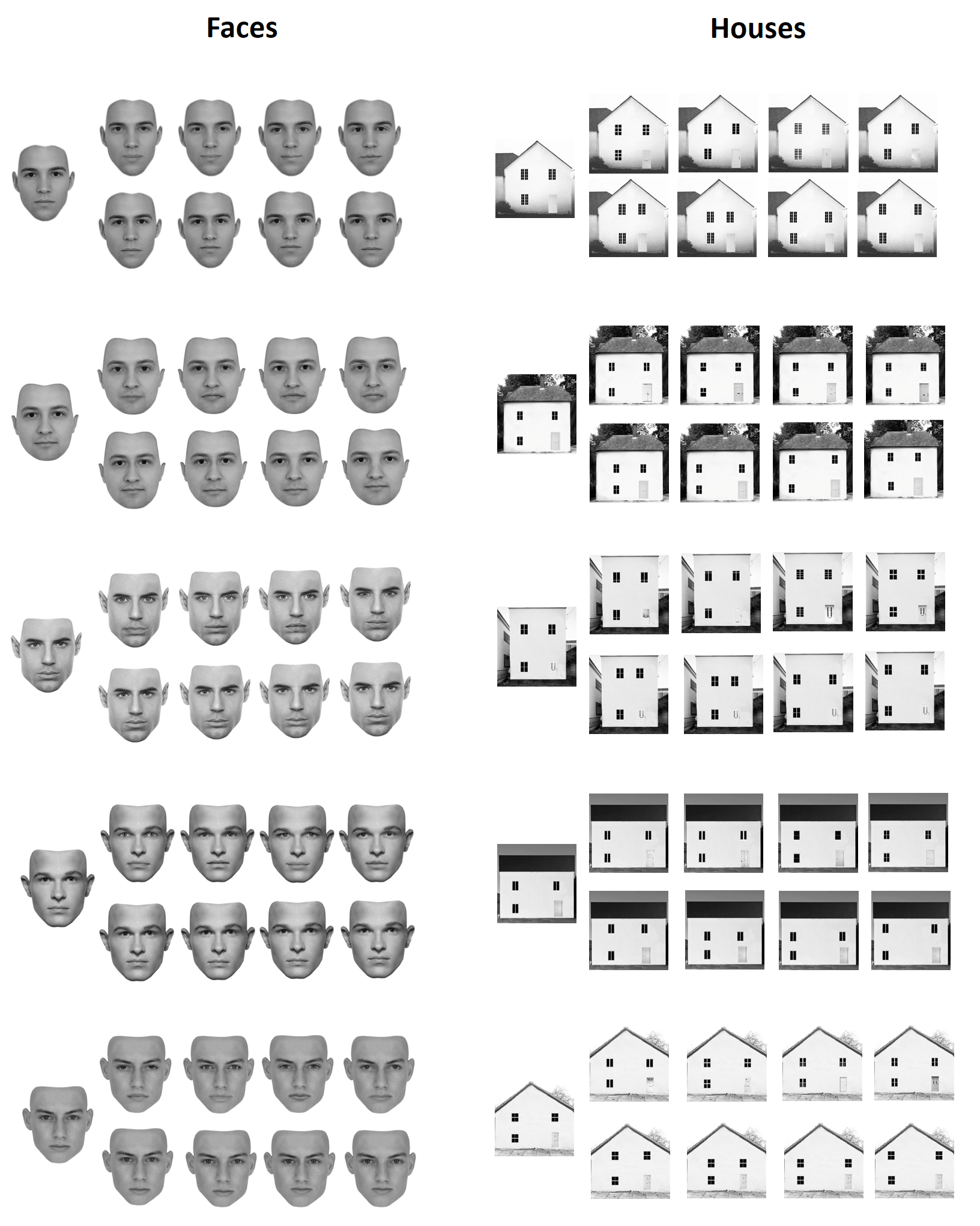

Supplement: Supplementary file 1 [file Image_1.TIF]
